# Cch1p Mediates Ca^2+^ Influx to Protect *Saccharomyces cerevisiae* against Eugenol Toxicity

**DOI:** 10.1371/journal.pone.0043989

**Published:** 2012-09-13

**Authors:** Stephen K. Roberts, Martin McAinsh, Lisa Widdicks

**Affiliations:** 1 Division of Biomedical and Life Sciences, Faculty of Health and Medicine, Lancaster University, Lancaster, United Kingdom; 2 Lancaster Environment Centre, Lancaster University, Lancaster, United Kingdom; Indiana University School of Medicine, United States of America

## Abstract

Eugenol has antifungal activity and is recognised as having therapeutic potential. However, little is known of the cellular basis of its antifungal activity and a better understanding of eugenol tolerance should lead to better exploitation of eugenol in antifungal therapies. The model yeast, *Saccharomyces cerevisiae*, expressing apoaequorin was used to show that eugenol induces cytosolic Ca^2+^ elevations. We investigated the eugenol Ca^2+^ signature in further detail and show that exponentially growing cells exhibit Ca^2+^ elevation resulting exclusively from the influx of Ca^2+^ across the plasma membrane whereas in stationary growth phase cells Ca^2+^ influx from intracellular and extracellular sources contribute to the eugenol-induced Ca^2+^ elevation. Ca^2+^ channel deletion yeast mutants were used to identify the pathways mediating Ca^2+^ influx; intracellular Ca^2+^ release was mediated by the vacuolar Ca^2+^ channel, Yvc1p, whereas the Ca^2+^ influx across the plasma membrane could be resolved into Cch1p-dependent and Cch1p-independent pathways. We show that the growth of yeast devoid the plasma membrane Ca^2+^ channel, Cch1p, was hypersensitive to eugenol and that this correlated with reduced Ca^2+^ elevations. Taken together, these results indicate that a cch1p-mediated Ca^2+^ influx is part of an intracellular signal which protects against eugenol toxicity. This study provides fresh insight into the mechanisms employed by fungi to tolerate eugenol toxicity which should lead to better exploitation of eugenol in antifungal therapies.

## Introduction

Fungi are emerging as major causes of human infections, particularly amongst a growing population of immunocompromised hosts, which is having significant economic and social impacts [1]. Our current situation is exacerbated by the limited number of antifungal drugs and the increasing incidence of resistance to (and failure of) antifungal treatments [1]. It is widely accepted that new therapeutic strategies are required.

Plant essential oils have been widely documented to possess broad spectrum antifungal properties and are generally recognised as safe for human and animal consumption [2]. Although plant essential oils have a complex chemical composition, phenolic compounds such as eugenol (the major constituent of essential oils from clove, cinnamon, and bay leaves), carvacrol (major constituent in oregano oil) and thymol (major constituent in thyme oil) have been identified as primary antimycotic components of essential oils and are recognised as having therapeutic potential [2], [3]. Possible modes of action to explain the antifungal capacity of these compounds have been suggested, including general disruption of membrane integrity and consequential disruption of cell signalling and leakage of cell contents [4]; however, the mechanism of killing is not clear and consequently we know nothing about the mechanisms employed by fungi to resist the antifungal properties of plant essential oils. Recently, Rao et al. [5] showed that a variety of phenolic compounds derived from plant essential oils induced cytosolic Ca^2+^ (Ca^2+^_cyt_) elevation in the model yeast, *Saccharomyces cerevisiae*. The authors specifically focussed on carvacrol and showed that this phenolic compound disrupted ion (Ca^2+^ and H^+^) homeostasis and induced transcriptional changes indicative of Ca^2+^ stress, raising the possibility that the antifungal activity of carvacrol depended, at least in part, on a toxic elevation of Ca^2+^. However, despite these new insights, it remains unclear if the Ca^2+^_cyt_ elevation induced by these plant phenolic compounds represents an antifungal activity or if it forms part of signalling response to protect against the fungicidal activity.

In the present study, we monitored aequorin luminescence to investigate the effects of eugenol on Ca^2+^ homeostasis of *S. cerevisiae* to gain insights into the mechanisms mediating its antifungal activity. We focussed on the role of the Ca^2+^ channels, Cch1p, Mid1p and Yvc1p and show that eugenol-induced Ca^2+^_cyt_ elevations are dependent on Cch1p-mediated Ca^2+^ influx. Furthermore, in contrast to that proposed for carvacrol, eugenol-induced Ca^2+^_cyt_ elevations do not appear to serve as a cytotoxic Ca^2+^ burst but instead are part of a signalling pathway which protects yeast against eugenol stress.

## Materials and Methods

### Strains and media

Single and double *mid1Δ* and *cch1Δ* mutants were derived from the parental *Saccharomyces cerevisiae* strain JK9-3da (Mata, leu2-3, 112, his4, trp1, ura 3–52, rme1, HMLa) by replacing the MID1 and CCH1 genes by a KanMX cassette [6]. *yvc1Δ* mutant was derived from the parental strain *S. cerevisiae* strain BY4742 (Matα, his3Δ1, leu 2Δ0, lys2Δ0, ura3Δ) by replacing YVC1 gene by a KanMX cassette (EUROSCARF strain Y11863). Unless otherwise stated, yeast strains were cultured at 30°C in standard synthetic complete media (SCM; Formedium, UK) or, for strains transformed with pEVP11/AEQ, SCM minus the addition of leucine (SCM-leu). All growth media contained 2% (w/v) glucose.

### Ca^2+^-dependent aequorin luminometry

Yeast strains were transformed with pEVP11/AEQ (a plasmid bearing apoaequorin gene and a LEU2 marker, generously provided by Dr Patrick Masson, University of Wisconsin-Madison, Wisconsin, US) as previously described [7]. To obtain cells in stationary growth phase, *S. cerevisiae* strains expressing apoaequorin were grown overnight in SCM-leu in a shaking (150 rpm) incubator to optical density at 600 nm (OD_600_) of 8 (1×10^8^ cells/ml). OD_600_ was determined after 1∶8 dilution of culture in water. To obtain cells in mid-logarithmic growth phase, 0.5 ml of stationary phase cells from overnight cultures were sub-cultured into 10 ml of fresh SCM-leu to give an OD_600_ of 0.8 and incubated at 30°C, shaking at 150 rpm for 4 to 5 hours until OD_600_ between 2.4 and 3.2 was reached. Cells were pelleted using a microcentrifuge and resuspended in fresh SCM-leu to an OD_600_ of 8.

Luminometry measurements were conducted as previously described [8]. Briefly, 20 µl of yeast cells (at 1×10^8^ cells/ml) transformed with pEVP11/AEQ were incubated with 0.5 µl of 0.5 mM coelentrazine (prolume, USA) in absolute methanol for 20 minutes in order to reconstitute functional aequorin with in the cells. After incubation, the base line luminescence was recorded every 0.2 seconds for 40 seconds (unless otherwise stated) using a digital chemiluminometer (Electron Tubes Ltd., UK). At 40 seconds, 200 µl of eugenol containing media was carefully added using a 1 ml syringe connected to a hypodermic needle. Luminescence (expressed in arbitrary units (AU) per 0.2 seconds) was measured for up to eight minutes after which cells were lysed with 1.6 M CaCl_2_ in 20% (v/v) ethanol to determine total (summed) luminescence. Comparing total luminescence indicated that aequorin production was similar in the strains used in the present study (data not shown), however, as previously reported by [9], total luminescence was greater (approximately two-fold) in logarithmically growing cells compared to stationary growth phase cells. Furthermore, total luminescence was in significant excess over luminescence induced by eugenol indicating that the availability of aequorin-coelentrazine complex was sufficient for the reporting of eugenol-induced Ca^2+^_cyt_ elevations. Samples were treated with coelentrazine sequentially maintaining a constant time of incubation before addition of eugenol. Eugenol (Sigma-Aldrich) was in liquid form (density 1.06 g/ml) and was made to 100× stocks in absolute ethanol and stored at 4°C. Eugenol was added to samples at indicated concentrations (containing 1% ethanol) in either SCM-leu (which contained 2% *w/v* glucose) or EGTA buffer (25 mM Na_2_EGTA, 10 mM HEPES, pH 7.4, 2% *w/v* glucose).

### Yeast toxicity Assays

Growth assays in liquid culture were conducted as follows: yeast strains were cultured overnight to OD_600_ of 8. 0.25 ml of cells were added to 5 ml of SCM (containing varying concentrations of eugenol and 1% ethanol) to a final OD_600_ of approximately 0.5 (6.6×10^6^ cells/ml). Cells were then incubated at 30°C shaking at 150 rpm. Controls contained 1% ethanol (solvent). At the time points indicated, aliquots were removed and OD_600_ determined.

For drop assays, yeast strains were grown overnight at 30°C in SCM to OD_600_ of 8, centrifuged, washed in 10 ml of sterile water and resuspended to 0.5×10^8^ cells/ml. Following 10-fold serial dilutions of each yeast suspension using sterile water, 5 µl drops were spotted on to SCM media (with varying concentrations of eugenol and 1% ethanol) containing 2% agar. Plates were incubated at 30°C.

## Results and Discussion

Rao et al. [5] showed that phenolic compounds from plant essential oils induce Ca^2+^_cyt_ elevations in *S. cerevisiae*. CCH1 and MID1 have been shown to encode subunits of a high affinity Ca^2+^ channel located in the yeast plasma membrane [10], [6], [11], [12]. Thus we explored the possibility that Cch1p and Mid1p mediated eugenol induced Ca^2+^ influx across the plasma membrane using Ca^2+^ channel mutant yeast strains transformed with pEVP11/AEQ [8] resulting in cytosolic expression of the Ca^2+^-sensitive bioluminescent protein aequorin. It has been recently shown that Ca^2+^ channel activity is dependent on the metabolic state of yeast cells [9], [13], [14] and thus Ca^2+^-dependent luminescence was monitored in cells in mid-logarithmic growth phase and in stationary growth phase.

### Eugenol-induced Ca^2+^_cyt_ elevations in mid-logarithmic growth phase cells

**Figure 1:**
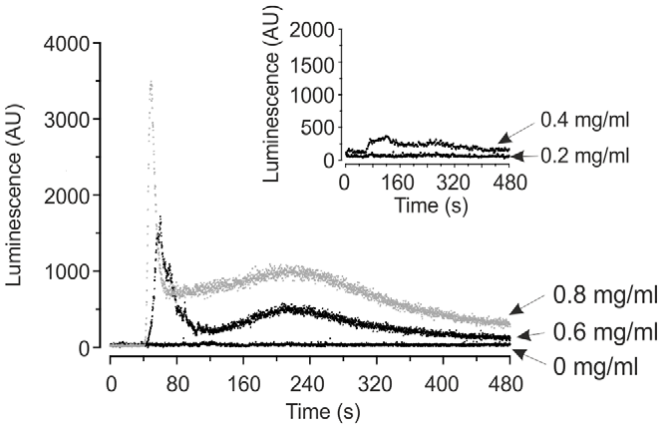
Eugenol Ca^2+^ signal in mid-log growth phase cells. Representative traces showing response of Ca^2+^-dependent aequorin luminescence in JK9-3da cells in mid-logarithmic growth phase to increasing concentrations of eugenol suspended in SCM-leu. Eugenol was added at 40 seconds. Luminescence was recorded every 0.2 second and is expressed in arbitrary units (AU). Inset: As main figure but with Ca^2+^-dependent luminescence recorded every 2 seconds.

Figure 1 shows representative traces of Ca^2+^-dependent luminescence in exponentially growing JK9-3da cells in response to increasing concentrations of extracellular eugenol. At concentrations greater than 0.4 mg/ml, eugenol induced a biphasic elevation in Ca^2+^_cyt_: a rapid transient increase in Ca^2+^_cyt_ is immediately elicited by eugenol followed by a more sustained increase in Ca^2+^_cyt_ which lasted several minutes, peaking at approximately 220 seconds. Elevations in Ca^2+^_cyt_ were difficult to resolve at concentrations less than 0.6 mg/ml, when recording luminescence every 0.2 seconds. However increasing the period over which the luminescence counts were integrated from 0.2 seconds to 2 seconds improved resolution and Ca^2+^_cyt_ elevations following addition of 0.4 mg/ml eugenol were consistently observed (n = 5) while increases in luminescence following addition of 0.2 mg/ml eugenol were not apparent (Figure 1 inset). A biphasic elevation in Ca^2+^_cyt_ in *S. cerevisiae* has also been reported in response to amiodarone [15]. In contrast, Rao et al. [5] reported a single Ca^2+^ peak in *S. cerevisiae* in response to carvacrol (0.0125–0.05 mg/ml), thymol (0.5 mg/ml) and eugenol (0.5 mg/ml) however, their recordings were limited to 80 seconds duration and therefore the biphasic nature of the response observed in the present study would not have been detected.

**Figure 2:**
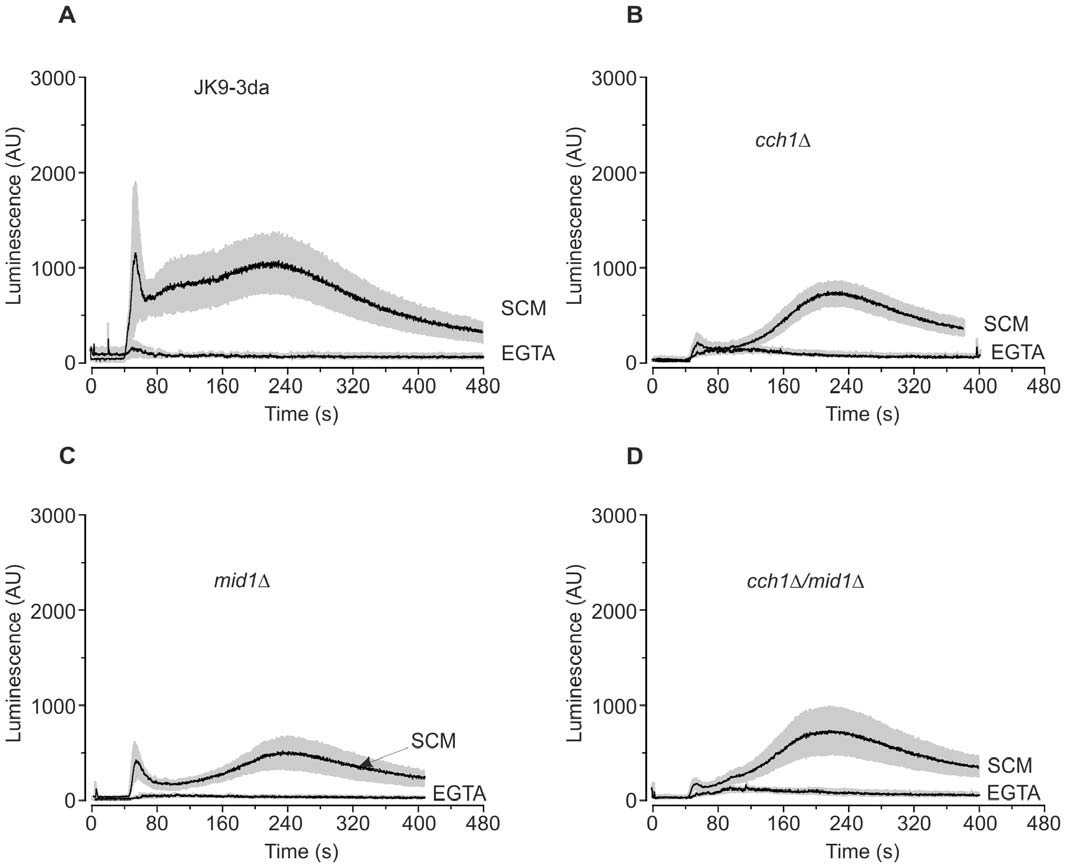
Eugenol induces Ca^2+^ influx across the plasma membrane in mid-log cells. Ca^2+^-dependent aequorin luminescence from JK9-3da (A), *cch1Δ* (B), *mid1Δ* (C) and *cch1Δmid1Δ* (D) cells in mid-logarithmic growth phase in response to 0.6 mg/ml eugenol. Eugenol was added at 40 seconds and was suspended in either SCM-leu (SCM) or EGTA buffer (EGTA). Traces represent mean (± SEM) from at least 5 independent experiments. SEM values are illustrated using grey shading. Luminescence was recorded every 0.2 seconds and is expressed in arbitrary units (AU).

In a bid to understand the contribution of extracellular and intracellular sources of Ca^2+^ in the eugenol response, Figure 2 shows average (± SEM) response of Ca^2+^_cyt_ following the application of 0.6 mg/ml eugenol in presence (i.e. SCM which contains approximately 0.1 mM Ca^2+^) and absence (i.e. EGTA buffer which is nominally Ca^2+^ free) of extracellular Ca^2+^. In the absence of extracellular Ca^2+^ no significant increase in Ca^2+^_cyt_ was detectable in the yeast strains tested indicating that the eugenol-induced Ca^2+^_cyt_ elevations resulted exclusively from an influx of Ca^2+^ across the plasma membrane. Interestingly, the eugenol-induced increase in Ca^2+^_cyt_ in yeast mutants devoid of cch1p (Figures 2B and D) was different to that in the parental and *mid1Δ* strains (Figures 2A and C) in that the transient increase in Ca^2+^_cyt_ which immediately follows the addition of eugenol was largely absent in mutant strains devoid of Cch1p. These results reveal that the eugenol-induced Ca^2+^_cyt_ elevation have two components; a cch1p-dependent Ca^2+^ influx which is activated immediately after addition of eugenol and a cch1p-independent Ca^2+^ influx which exhibits a delayed and more sustained activity lasting several minutes. This is consistent with the proposal that there are functionally redundant Ca^2+^ entry pathways in *S. cerevisiae* that remain to be identified [16]. It is also noteworthy that the eugenol-induced cch1p-dependent Ca^2+^_cyt_ increase in *mid1Δ* cells was reduced compared to the parental strain. The first peak of the amiodarone-induced Ca^2+^ elevation has also been shown to originate from Ca^2+^ influx across the plasma membrane, although there are contrasting reports as to the influx pathways involved and the effects of *cch1Δ* and *mid1Δ* on the Ca^2+^_cyt_ increase [13], [15], [17], whilst the second peak is predominantly derived from Ca^2+^ influx from the vacuolar store via the TRP-like Ca^2+^ channel Yvc1p [15], [18]. Taken together, our results are consistent with the notion that eugenol activates Cch1p to elicit a Ca^2+^ influx across the plasma membrane and that Mid1p is necessary for optimal Cch1p activity.

### Eugenol-induced Ca^2+^_cyt_ elevations in stationary growth phase cells

**Figure 3:**
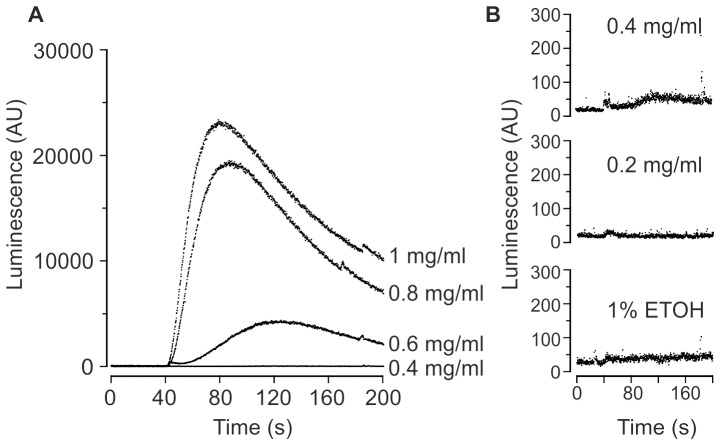
Eugenol Ca^2+^ signal in stationary growth phase cells. A) Representative traces showing response of Ca^2+^-dependent aequorin luminescence in JK9-3da cells in stationary growth phase to increasing concentrations of eugenol suspended in SCM-leu. Eugenol was added at 40 seconds. Luminescence was recorded every 0.2 second and is expressed in arbitrary units (AU). B) data from the same experiment shown in part A but expressed on an expanded y-axis scale.

**Figure 4:**
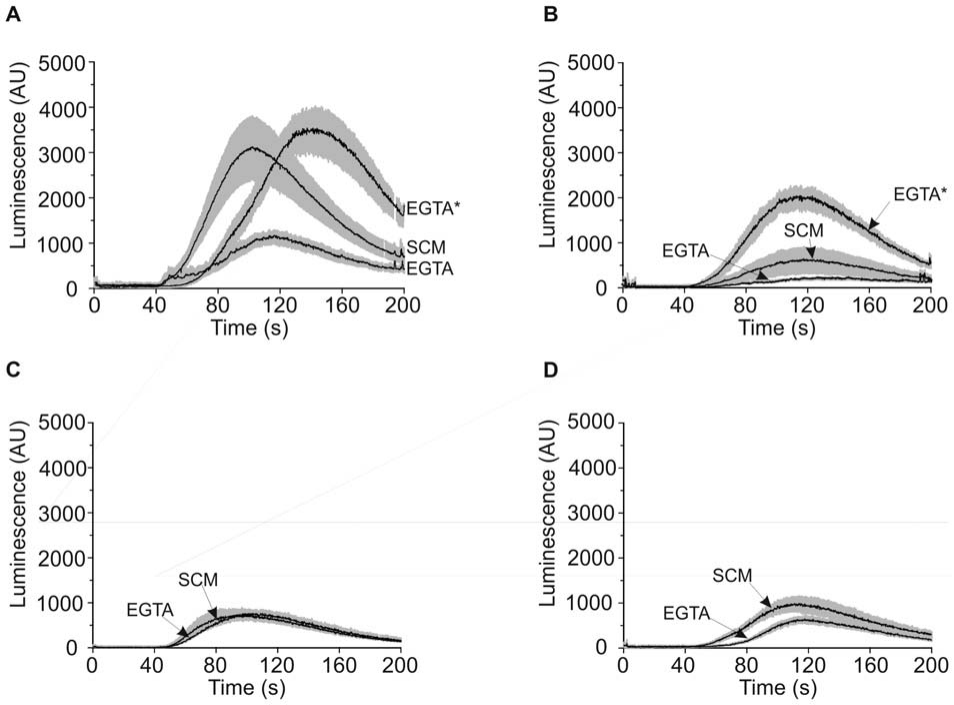
Eugenol induces Ca^2+^ influx from intracellular and extracellular Ca^2+^ sources in stationary phase cells. Ca^2+^-dependent aequorin luminescence from JK9-3da (A), *cch1Δ* (B), *mid1Δ* (C) and *cch1Δmid1Δ* (D) cells in stationary growth phase in response to 0.6 mg/ml eugenol. Eugenol was added at 40 seconds and was suspended in either SCM-leu (SCM) or EGTA buffer (EGTA). EGTA* represents luminescence from JK9-3da and *cch1Δ* cells cultured overnight in SCM-leu supplemented with 10 mM CaCl_2_. Traces represent mean (± SEM) from at least 4 independent experiments. SEM values are illustrated using grey shading. Luminescence was recorded every 0.2 seconds and is expressed in arbitrary units (AU).

Previous reports have highlighted that Ca^2+^ signatures are dependent on the growth phase of yeast cells [9], [13], [14] and therefore we investigated eugenol-induced Ca^2+^_cyt_ elevations in stationary growth phase cells. Figure 3 shows that the eugenol-induced Ca^2+^ elevation in stationary phase cells was distinct to that exhibited by mid-logarithmic growth phase cells (Figure 2); specifically, stationary phase cells exhibited only one discernible phase which peaked within 120 seconds (in a concentration-dependent manner) and the magnitude of the Ca^2+^_cyt_ elevation was up to 10-fold greater. Eugenol-induced increases in Ca^2+^-dependent luminescence were detectable in stationary growth phase cells at eugenol concentrations of 0.4 mg/ml and above (Figure 3B). We investigated the eugenol-induced Ca^2+^ responses in further detail and average (±SEM) responses to 0.6 mg/ml eugenol are shown in Figure 4. In the absence of extracellular Ca^2+^, the eugenol-induced Ca^2+^_cyt_ elevations in the parental strain (JK9-3da) were reduced by approximately 65% indicating that, in contrast to cells in mid-logarithmic growth phase, eugenol induces Ca^2+^ influx from both extracellular and intracellular sources (Figure 4A). However, extracellular Ca^2+^ influx was significantly reduced and Ca^2+^ release from intracellular stores was completely abolished in *cch1Δ* cells (Figure 4B). These results indicated that CCH1 was necessary for Ca^2+^ influx across the plasma membrane and influenced Ca^2+^ release from intracellular stores. It has been previously reported that deletion of CCH1 reduces the intracellular Ca^2+^ content in yeast and as a result, exhibit reduced intracellular Ca^2+^ release in response to hyperosmotic shock [9]; this is consistent with the notion that Cch1p functions to replenish intracellular stores [11], [19], [12]. We investigated the possibility that the absence of eugenol-induced Ca^2+^ release from intracellular stores in *cch1Δ* mutants also resulted from an inability to maintain intracellular Ca^2+^. JK9-3da and *cch1Δ* yeast strains were cultured in SCM-leu supplemented with 10 mM CaCl_2_ to promote accumulation of intracellular Ca^2+^. The Ca^2+^-replete cells of wild type and *cch1Δ* strains exhibited significantly greater (approximately 4-fold) release of intracellular Ca^2+^ following addition of eugenol (Figures 4A and B) indicating that Cch1p is necessary to maintain intracellular Ca^2+^ stores in the absence of high extracellular Ca^2+^. Interestingly the eugenol-induced intracellular Ca^2+^ release from *mid1Δ* and *cch1Δmid1Δ* strains (Figures 4 C and D) was comparable to the wild type strain suggesting that in the absence of Mid1p, yeast may employ mechanisms to maintain intracellular Ca^2+^ stores independently of Cch1p. Indeed, Mid1p has been proposed to act in sensing intracellular Ca^2+^ and couple Cch1p activity to intracellular Ca^2+^ content [12]; thus it is tempting to speculate that *mid1Δ* yeast adopt alternative (hitherto unidentified) pathways to maintain intracellular Ca^2+^.

**Figure 5:**
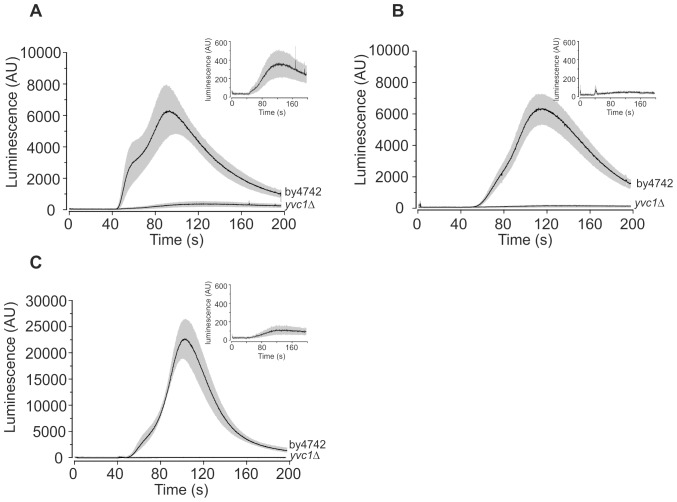
Yvc1p mediates intracellular Ca^2+^ release from stationary phase cells. Ca^2+^-dependent aequorin luminescence from BY4742 and *yvc1Δ* cells in stationary growth phase in response to 0.6 mg/ml eugenol. Eugenol was added at 40 seconds and was suspended in either SCM-leu (A) or EGTA buffer (B). Traces represent mean (± SEM) from at least 4 independent experiments. SEM values are illustrated using grey shading. Luminescence was recorded every 0.2 seconds and is expressed in arbitrary units (AU). Inset shows data for *yvc1Δ* cells except expressed on an expanded y-axis scale. C) As part (B) except cells were cultured overnight in SCM-leu supplemented with 10 mM CaCl_2_.

**Figure 6:**
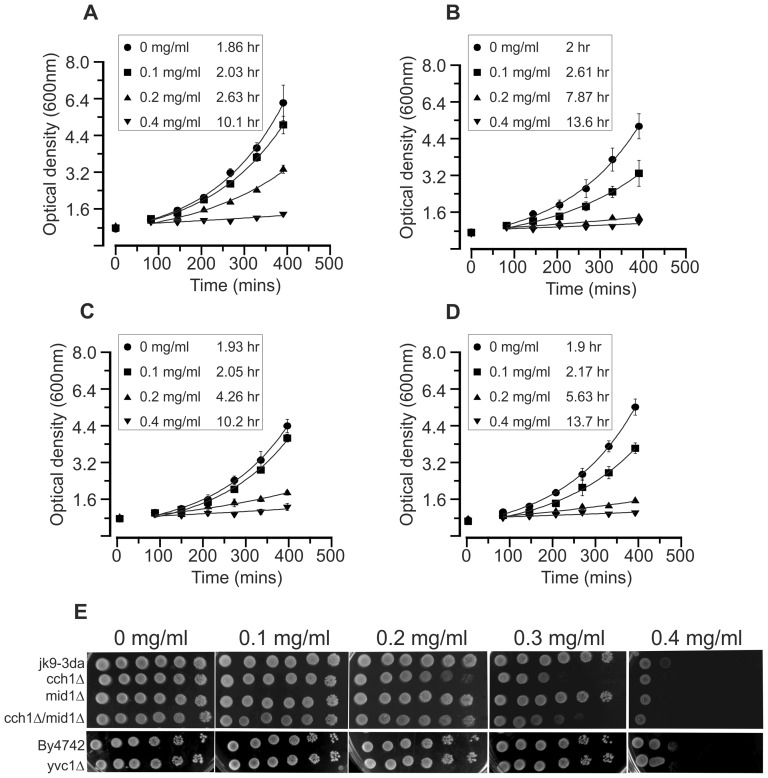
CCH1 is necessary for eugenol tolerance. Growth of JK9-3da (A), *cch1Δ* (B), *mid1Δ* (C) and *cch1Δmid1Δ* (D) yeast in response to increasing concentrations of eugenol. Growth was in liquid SCM at 30°C (shaking at 150 rpm). Doubling times are shown in boxes for each strain. Doubling times were calculated from fits of the data in the exponential phase of growth. Fits are to number of cells (OD_600 nm_) = OD_600 nm_ at time 90 minutes x e^(growth rate x time)^ were growth rate is the number of doublings per minute. Data are the mean (± SD) of at least three independent experiments. E) Yeast culture was spotted onto SCM agar plates containing 0 (1% ethanol), 0.1, 0.2, 0.3 and 0.4 mg/ml eugenol. Left most spots on each plate are growth after 3 days at 30°C after inoculation with 5 µl culture at approximately 0.5×10^8^ cells/ml. Serial 10-fold dilution of the first innocula are shown to the right.

To investigate the origin of the eugenol-induced intracellular Ca^2+^ release, the *yvc1Δ* mutant and the isogenic parental strain (BY4742) were transformed with pEVP11/AEQ. Yvc1p is a TRP-like Ca^2+^ release channel [18] that mediates Ca^2+^ influx from the vacuole in response to osmotic shock [20]. Figure 5 shows the average (±SEM) eugenol-induced Ca^2+^_cyt_ elevations in *yvc1Δ* and BY4742 (the isogenic parental strain) cells in stationary growth phase. Surprisingly, the magnitude of the eugenol-induced Ca^2+^_cyt_ elevations in BY4742 cells was similar in both the absence and presence of extracellular Ca^2+^ (compare Figures 5A and B) indicating that, in contrast to the JK9-3da strain, intracellular release of Ca^2+^ is the major component of the eugenol response in stationary BY4742 cells. Consistent with this, eugenol-induced Ca^2+^_cyt_ elevations in *yvc1Δ* cells were small in the presence of extracellular Ca^2+^ (Figure 5A) and completely abolished in EGTA buffer (Figure 5B). However, despite the relatively small contribution of the extracellular component of the Ca^2+^ signal in BY4742 cells, it is noteworthy that the Ca^2+^ influx across the membrane is evident in that the onset of the Ca^2+^ increase is earlier and increases more rapidly in the presence of extracellular Ca^2+^ (Figure 5A) compared to that in the absence of extracellular Ca^2+^ (Figure 5B). As expected, overnight culture in SCM-leu supplemented with 10 mM CaCl_2_ resulted in enhanced eugenol-induced intracellular Ca^2+^ release in the parental strain (BY4742) but was absent in the *yvc1Δ* mutant (Figure 6C). Taken together, these results show that eugenol activates Yvc1p to mediate Ca^2+^ release from the vacuole in stationary cells.

The differences in the Ca^2+^ response to eugenol between stationary growth phase yeast and logarithmically growing yeast are interesting and most likely reflect differences in Ca^2+^ channel activity as a result of the cells being in different metabolic states. This is consistent with previous reports. For example, the absence of Yvc1p activation (in response to osmotic shock) has been previously reported in logarithmically growing yeast cells [9] and the magnitude and the temporal kinetics of the Ca^2+^_cyt_ elevation in response to amiodarone are reduced in stationary phase cells compared to that exhibited in actively growing cells [13].

### Eugenol toxicity assays

To distinguish between the possibilities that Ca^2+^ influx and Ca^2+^_cyt_ elevation are necessary for eugenol antifungal activity or form part of a tolerance mechanism we examined the growth of *cch1Δ* and *mid1Δ* single and *cch1Δmid1Δ* double mutants in increasing concentrations of eugenol (Figure 6). As shown in Figures 6A to D, the growth of *cch1Δ*and *cch1Δmid1Δ* strains in liquid SCM (characterised by doubling times of 7.87 and 5.63 hours respectively in the presence of 0.2 mg/ml eugenol) was more sensitive to eugenol relative to that for the isogenic parental strain (doubling time of 2.63 hours in 0.2 mg/ml eugenol). Although the growth of the *mid1Δ* strain (doubling time of 4.26 hours in 0.2 mg/ml eugenol) was marginally more sensitive to eugenol compared to the parental strain, inhibition of *mid1Δ* growth was less than that exhibited by yeast strains devoid of Cch1p. The hypersensitivity of the *cch1Δ* and *cch1Δmid1Δ* mutant strains to eugenol could be also clearly demonstrated in drop assays on solid SCM (Figure 6E). The drop assay also confirmed that the *mid1Δ* mutant strain was more tolerant to eugenol than the strains devoid of Cch1p. Taken together these results indicated that Cch1p-mediated Ca^2+^ influx is most likely part of a Ca^2+^-dependent signal which protects against the toxic effects of eugenol. Interestingly, the growth of the *yvc1Δ* mutant was equivalent to its isogenic parental strain, BY4742 (Figure 1E) indicating that the Ca^2+^ influx from intracellular stores was not involved in the response of yeast to eugenol.

Figure 6 also indicates Cch1p activity in the absence of Mid1p. It has been well documented that CCH1 and MID1 are necessary for yeast survival in response to ion stress and azole class antifungals [12], [19], [21], [22], [23]; in these studies, *mid1Δ* and *cch1Δ* single and double mutants displayed the same phenotype and support the notion that Mid1p and Cch1p are subunits of the same channel. The dependency of Cch1p function on Mid1p has also been shown in electrophysiological experiments [12] in which heterologously-expressed Cch1p activity was only apparent with the co-expression of Mid1p. However, the present study shows that Cch1p can function independently of Mid1p which has been also been reported in *S. cerevisiae* in response to temperature and ion stress [24]. In addition, *Candidia albicans* MID1 appears to play a more prominent role in thigmotropism while CCH1 was more important for galvanotropism [25]. Thus it appears that under certain conditions, Cch1p and Mid1p can function independently.

### Insights into eugenol tolerance

In the present study, actively growing cells are inhibited by eugenol (Figure 6) and hence the Ca^2+^ response to eugenol in yeast cells in logarithmic growth phase is of most interest when considering the antifungal activity of eugenol. Clearly CCH1 plays a role in eugenol tolerance. *Cch1Δ* mutants exhibit hypersensentive growth to eugenol and this correlates with a reduction in Ca^2+^_cyt_ elevation indicating that the Cch1p-mediated Ca^2+^ influx is probably part of a signal response to protect the cells during eugenol stress. Interestingly, addition of 2 µg/ml of FK506 had no effect on the growth of JK9-3da or *cch1Δ* strains in the presence of eugenol (experiments conducted as for Figure 1A; data not shown) indicating that the eugenol response signal does not involve the activation of calcineurin. The mechanism for Cch1p activation by eugenol is currently unknown.It is noteable that the Cch1p-mediated Ca^2+^ elevation is activated immediately following addition of eugenol and thus raises the possibility that Cch1p is directly activated by eugenol. However, eugenol has been shown to generate reactive oxygen species (ROS) in animal cells [28] and ROS (namely H_2_O_2_) induces Ca^2+^ elevations in yeast [29], [30]. Thus it will be interesting in future studies to elucidate the activation of Cch1p by eugenol in more detail and determine if eugenol activates Cch1p via the generation ROS. From the present study, it is apparent that eugenol-induced Cch1p activity is not dependent on Mid1p. γ-subunits are known to bind and regulate Cch1-like α-subunits of voltage-gated calcium channels in animal cells. Recently, a γ-subunit homolog, Ecm7p, has been identified in *S. cerevisae* which appears to regulate Cch1p-mediated Ca^2+^ influx [31]. Thus, it will be interesting to determine if eugenol activation of Cch1p in yeast is also dependent on Ecm7p.

It is also noteworthy that the elevation of Ca^2+^_cyt_ is unlikely to represent a toxic Ca^2+^ burst resulting in cell death. For example, there is no correlation between the inhibition of growth in different yeast strains and the magnitude of Ca^2+^_cyt_ increase induced by eugenol. It is also noteworthy that the concentration of eugenol required to inhibit yeast growth (0.2 to 0.4 mg/ml) overlapped with modest increases in Ca^2+^_cyt_ (consistent with the Ca^2+^ elevations representing a cytosolic signal) and were below that required to induce large Ca^2+^_cyt_ elevations (which are more likely to represent a toxic burst of Ca^2+^).

The antifungal mechanism of eugenol appears to be distinct to that reported for amiodarone, azoles and carvacrol. Amiodarone toxicity in yeast has been extensively studied and most lines of evidence points towards a drug-induced calcium influx which constitutes a toxic Ca^2+^ burst [13], [27]. Notably, the amiodarone induced Ca^2+^ influx does not appear to involve Cch1p or Mid1p [13]; [ however also see 17] but rather the Ca^2+^ influx results from Ca^2+^ channels (of unknown molecular identity) which are activated by amidorane-induced membrane hyperpolarisation [27]. Furthermore, in contrast to eugenol, amiodarone and azole tolerance is dependent on calcineurin [15], [26]. Much less is known about the mechanisms mediating carvacrol toxicity, however based on the similarities between the transcriptional response to amiodarone and carvacrol, Rao et al. [5] proposed that carvacrol elicits Ca^2+^ stress and Ca^2+^-mediated cell death. It would therefore be interesting to test the sensitivity of *cch1Δ* and *mid1Δ* mutant growth to carvacrol.

Future studies should focus on elucidating the signalling pathway (downstream of the Ca^2+^ signal) conferring eugenol tolerance in yeast. It will also be interesting to identify the channel(s) mediating Cch1p-independent Ca^2+^ influx in response to eugenol and determine if these channels contribute to eugenol tolerance. This will improve our understanding of eugenol toxicity which should lead to better exploitation of eugenol in antifungal therapies.
